# Imaging Characteristics and Differential Diagnosis of Newborn Oral Tumors Based on a Prenatally Diagnosed Neumann Tumor

**DOI:** 10.3390/diagnostics16193131

**Published:** 2026-09-26

**Authors:** Krisztián Nagy, Zsuzsanna Nagy, Agnes Kothencz, Gergő Leipold, Gergő Göbl, Tibor Glasz, Piotr Kuropka, Ömer Uranbey, Andrea Lakatos, Kamil Nelke

**Affiliations:** 1 Pediatric Center, Semmelweis University, 1083 Budapest, Hungary; 2 Department of Obstetrics and Gynecology, Semmelweis University, 1083 Budapest, Hungaryleipold.gergo@semmelweis.hu (G.L.); 3 Institute of Anesthesiology and Perioperative Care, Semmelweis University, 1083 Budapest, Hungary; gobl.gergely@semmelweis.hu; 4 Department of Pathology, Forensic and Insurance Medicine, Semmelweis University, 1083 Budapest, Hungary; 5 Division of Histology and Embryology, Department of Biostructure and Animal Physiology, Wrocław University of Environmental and Life Sciences, Cypriana K. Norwida 25, 50-375 Wrocław, Poland; 6 Department of Oral and Maxillofacial Surgery, Faculty of Dentistry, Aydın Adnan Menderes University, 09100 Aydın, Türkiye; 7 Diagnostic Imaging Center, Borsod-Abaúj-Zemplén County Central Hospital and University Teaching Hospital, Szentpéteri kapu 72–76, 3526 Miskolc, Hungary; 8 Maxillo-Facial Surgery Ward, EMC Hospital, Pilczycka 144, 54-144 Wrocław, Poland

**Keywords:** congenital epulis, Neumann tumor, alveolar bone, newborn, oral tumors

## Abstract

The spectrum of congenital lesions in newborns includes various soft and hard tissue masses found within or near the oral cavity. While oral clefts are often easily diagnosed prenatally and treated after birth, other critical conditions may require immediate neonatal intervention. These include airway compromise resulting from choanal atresia, alveolar mucosal fusion, or space-occupying lesions that reduce airway volume. Furthermore, some lesions may cause significant bleeding or interfere with sucking and feeding. In such cases, careful prenatal evaluation using ultrasound (USG) and magnetic resonance (MR) imaging is essential to inform the timing and strategy of surgical interventions. The occurrence of a rare lesion, such as a congenital epulis, also known as a Neumann tumor (NT), requires precise diagnostics to exclude other worrisome pathologies. Detailed MR evaluation of the oral commissure, tongue, lips, and gingival area helps identify the lesion’s extent and its anatomical relationship with adjacent structures. Although serial ultrasonography demonstrated interval growth, near-term MRI showed no pharyngeal extension or laryngotracheal narrowing. An EXIT-to-airway procedure was not planned, and delivery was performed by elective cesarean section with multidisciplinary airway support available. This report highlights that local NT removal typically focuses on complete excision, evaluation of the tumor base, and assessment of the surrounding alveolar bone.

**Figure 1:**
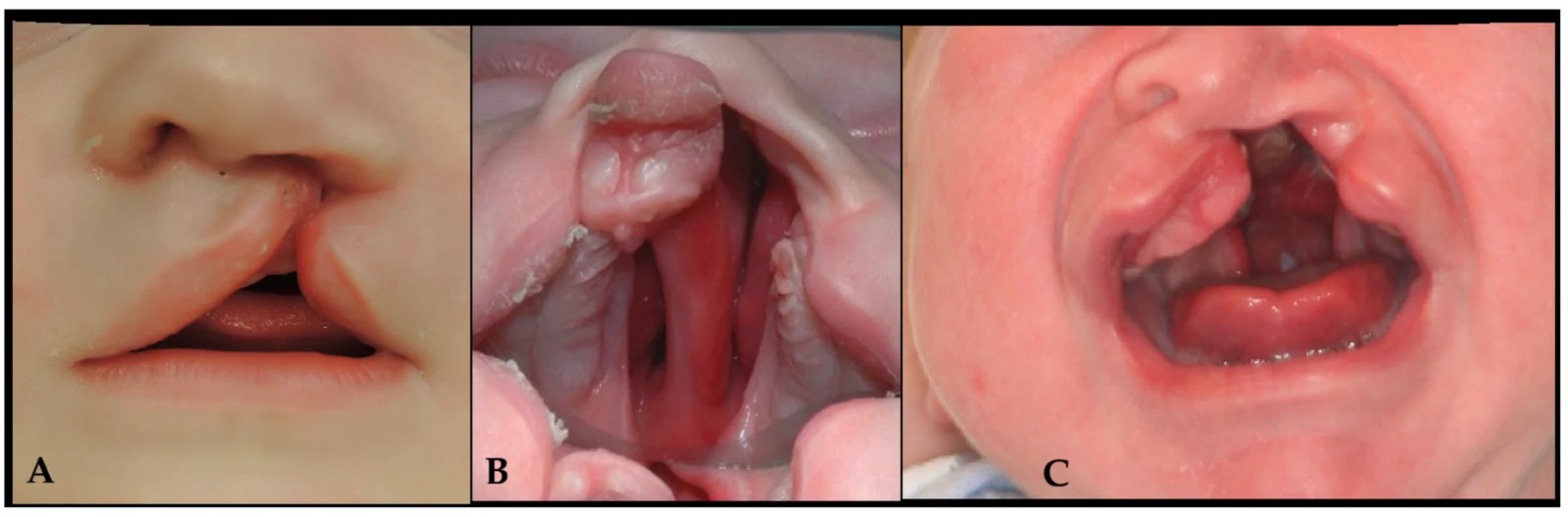
There are several known and common oral cavity and facial congenital anomalies. We can distinguish those arising from soft and hard tissues of the oral cavity, as well as both oral and facial clefts. During initial fetal ultrasound (USG) evaluation, some anatomical abnormalities can be readily identified. Both clefts and some neonatal tumors and anomalies can be found during clinical prenatal and pre-birth USG examinations. Oral clefts (**A**–**C**) are commonly identified in routine prenatal USG or based on the parents’ history [1,2]. (**A**–**C**) presents the most typical and most commonly found clefts in newborns. (**A**) Unilateral cleft lip, affecting only the soft tissue of the lip and the nostril. (**B**) Bilateral cleft lip and palate, with a prominent premaxilla. (**C**) Unilateral cleft lip and palate, with soft tissue and bony defects, where quite often the maxillary segments are misaligned, and the nasal base is dislocated. In those cases, there is a septal deviation and alar flaring. The three maxillary segments are displaced, and there is a broad alveolar and palatal cleft. The orbicular oris muscle is located in the lateral lip segments, which can cause dysfunction. The prolabium and the columella are short. Each of those anatomical features should always be assessed individually in each case. While the oral clefts shown in (**A**–**C**) represent common structural defects, they must be carefully differentiated from exophytic masses like the Neumann tumor during prenatal screening. Distinguishing between a primary cleft and a space-occupying tumor is essential for determining the risk of airway obstruction at birth. A different situation arises if, on USG, some worrisome exophytic or endophytic lesions within the oral cavity and face can be identified. The extent of those lesions and their anatomical relation to the skin, bone, vascular supply, and possible effects on the airway might quite substantially correlate with the necessity of an early or delayed surgical approach in the newborn. When a suspicious lesion is found during initial prenatal scanning, an MR (magnetic resonance) evaluation is one of the most commonly performed diagnostic tests to evaluate the fetus and the lesion. There are a vast number of possibilities within tumors found in newborns, and they can include: teratomas, neuroblastomas, hemangiomas, mucoceles, ranula, gingival cysts, Epstein pearls, Bohn’s nodules, eruption cysts, epidermoid/dermoid cysts, lymphatic malformations, lingual thyroid, oral choristomas, and other rare lesions [3,4]. One of the rarest fetal tumors and anomalies in newborns, which can be misdiagnosed with other pathologies, is a Neumann tumor (NT), also known as the congenital granular cell tumor (CGCT) [5,6]. NT is a rare finding in newborns and was first described by Neumann in 1871 as a benign soft-tissue lesion arising from the alveolar part of the maxillary bones or, rarely, from the mandible gingiva [1,2,3,4]. This epulis-like lesion is quite commonly found in the anterior area, mostly of single origin, whereas its multifocal appearance is very atypical and unique [4,5,6,7]. The origins and etiology of NT are still not fully understood, but it is mainly associated with the neonatal anterior gingival area, mostly in females. The alveolar ridge area should be closely investigated to confirm or exclude the presence of any other bone lesions or the spread of the NT inside the periosteum or within the jawbone structure. Because of significant gingival swelling, the entire surrounding area should be closely evaluated after excision. Malignant transformation has not been reported, and continued postnatal growth is considered uncommon; spontaneous regression of small lesions has also been described.

**Figure 2:**
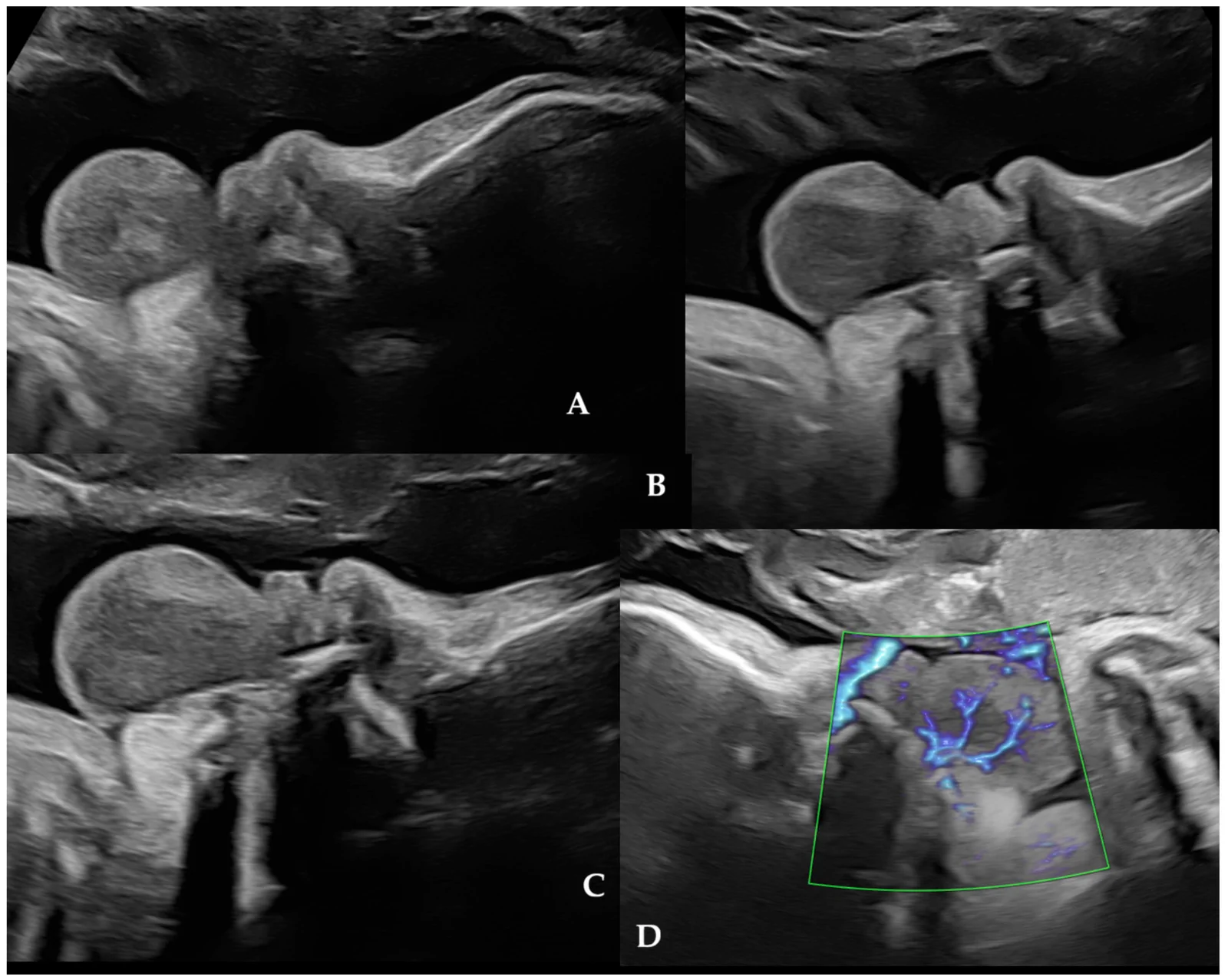
This article primarily focuses on the imaging characteristics and differential diagnosis of congenital oral masses, using a prenatally diagnosed Neumann tumor as the central illustrative example. Figure 2, Figure 3, Figure 4, Figure 5 and Figure 6 present the same newborn, including prenatal imaging, perinatal planning, postnatal clinical findings, surgical treatment, and histopathological confirmation. Ultrasound examination (USG: GE Healthcare, Chicago, United States) was conducted routinely (**A**–**C**) prenatally. Atypical structures were detected during routine ultrasonographic examination at 30 weeks of gestation, raising the suspicion of a congenital mass occupying the oral cavity. Therefore, fetal MRI was performed at 32 weeks and 3 days of gestation for further characterization. In this case, at 34 weeks of gestation, the lesion was described on USG as a tumor filling the oral opening, currently measuring 35 × 28 × 21 mm, with a slightly inhomogeneous structure, and showing clear vascularization. (**D**) The green area marks the area with increased vascular supply. USG is a very fast, low-cost, and highly effective screening tool for NT and other similar pathologies. At 36 weeks of gestation, another USG concluded that the tumor size had increased and measured 44 × 28 × 27 mm, which prompted a second fetal MRI near-term for more accurate assessment and perinatal planning. In each case of USG, when the result is not clear and the boundaries and adjacent tissue structures close to the lesion are hard to define, an MRI should improve diagnostics. Besides congenital epulis (Neumann tumor), other congenital oral or oropharyngeal lesions that may cause significant airway obstruction at birth include epignathus/oropharyngeal teratoma, extensive lymphatic or vascular malformations, and large cystic lesions of the tongue or floor of the mouth. These conditions may require immediate airway stabilization and, in selected prenatally diagnosed cases, consideration of an EXIT procedure (ex utero intrapartum treatment) [4,5,6,7,8,9,10,11,12].

**Figure 3:**
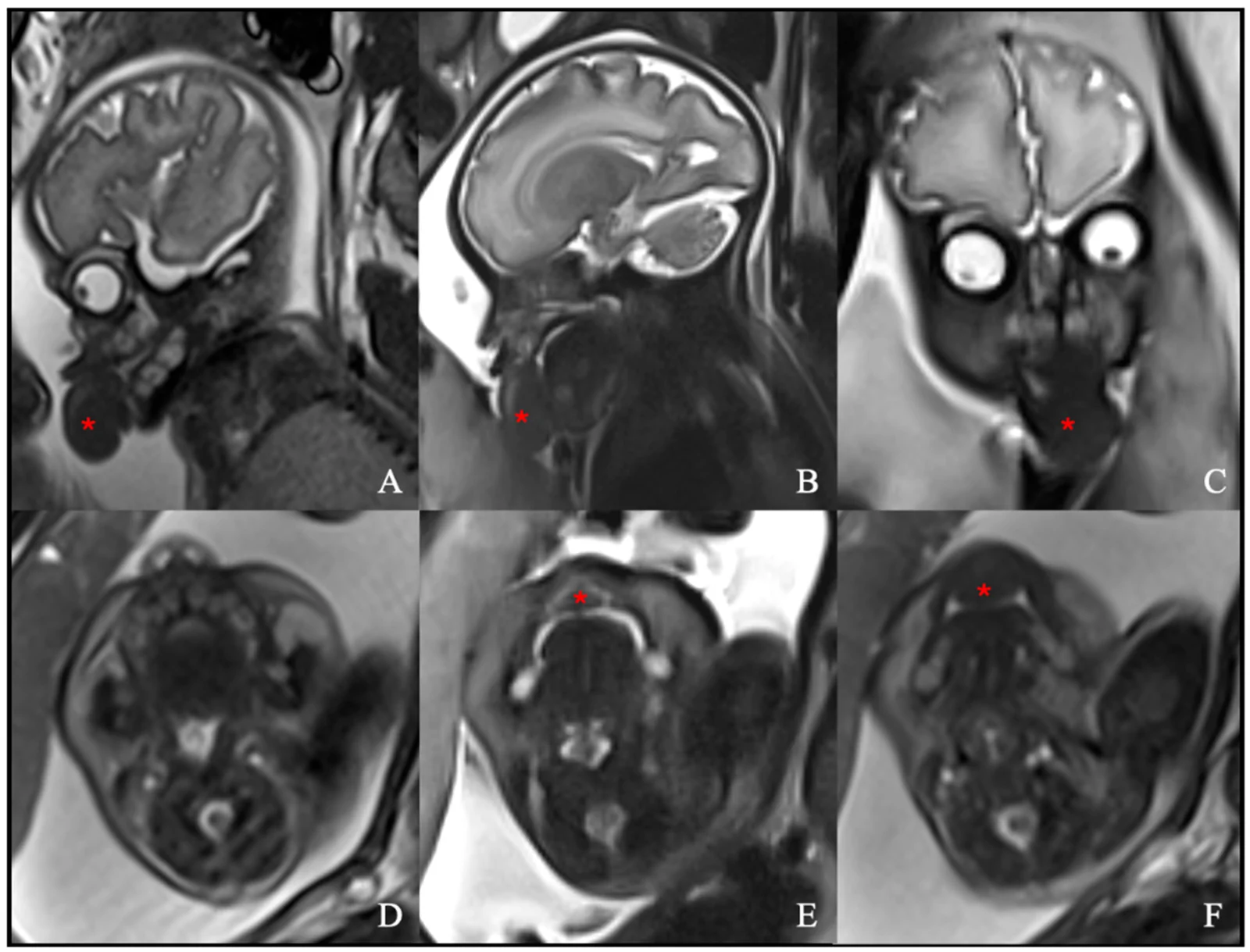
The Neumann tumor (NT) is a congenital granular cell tumor (CGCT) of the newborn, also known as congenital epulis or soft-tissue oral tumor, found in less than 0.05% of all newborn tumors (NBT) [5,6]. Some studies report that maxillary neonatal epulis is more than three times more common than the mandibular form [4,5,6,7]. CGCT most frequently involves the anterior mucosal alveolar region of the maxilla, whereas the mandible is a less typical site. Lesions are usually solitary, with variable shape and size, while multiple epulides are rare and may cause significant bleeding, ventilation problems, feeding difficulties, or local oral commissure dysfunction. When an oral mass is identified, surgical removal under local or general anesthesia is typically recommended; simple excision with coagulation is usually sufficient, and recurrence or lesion progression was not reported [7,8,9,10]. Ultrasound examination may reveal irregularities at the superficial aspect of the lips and the anterior parts of alveolar ridges, but it is limited in defining deeper soft-tissue relationships, exact lesion extent, and potential airway compromise. In order to improve diagnostic accuracy and assess asymmetry, exophytic lesion size, shape, configuration, and position, as well as tumor relationship to the tongue, palate, jaws, and upper airway, magnetic resonance imaging (MRI) should be carefully planned. (**A**–**F**) presents fetal MR imaging performed on a 3-Tesla Siemens Magnetom Skyra. The figure shows the fit system (Siemens Healthineers, Erlangen, Germany) at 32 weeks and 3 days of gestation. The maternal pelvis was examined in three planes using T2-weighted HASTE sequences; the fetal skull in three planes with T2-weighted HASTE and TRUFI, axial DWI and DTI, axial T1 VIBE FS, axial and coronal hemorrhage-sensitive sequences; and the fetal thoracoabdominal region in three planes with T2-weighted TRUFI, coronal DWI-STIR, and coronal and sagittal T1 VIBE FS. Sagittal T2-weighted TRUFI CINE sequences were acquired to assess fetal swallowing. The MRI demonstrated a well-circumscribed soft-tissue mass at the level of the oral opening ((**A**–**F**); asterisk), located in the midline and slightly off-center, with predominant extraoral protrusion, measuring approximately 15 × 30 × 30 mm. The lesion was inseparable from the lips (**A**,**B**) and showed a small intraoral component situated anterior to the tongue (**E**,**F**). The tongue appeared normal and showed no continuity with the lesion (**B**). Beyond partial obliteration of the oral opening, the exophytic mass did not cause significant narrowing within the oral cavity (**B**) and showed no extension toward the pharynx. A slight S-shaped deviation of the nasal septum was observed, while no cleft palate could be identified. The arches of the maxilla (**D**) and mandible (**E**) were intact and could be followed continuously. The pharynx and larynx, as well as the trachea and esophagus, appeared intact. The swallowing mechanism was normal on CINE (motion-capture) sequences. The mass exhibited homogeneous low-signal intensity on T2-weighted images, without evidence of cystic change, fat content, or calcification. Apart from the described lesion, no additional structural abnormalities were identified. Based on its imaging characteristics, the oral soft-tissue lesion was considered most compatible with a granular cell tumor (Neumann tumor). According to serial ultrasound examinations, progressive growth of the lesion raised concern for potential airway obstruction at birth. At 37 weeks and 1 day of gestation, follow-up fetal MRI confirmed a size-progressive soft-tissue mass with the same morphological characteristics and anatomical relationships as observed on the earlier MRI examination. Because ultrasound demonstrated increased vascularization of the lesion, potential connections with facial, labial, or submental arteries and the associated risk of hemorrhagic complications had to be anticipated, while MRI was of limited value in confirming these vascular relationships compared with ultrasound. An EXIT-to-airway procedure was not planned. The prenatal imaging findings relevant to this management included a predominantly extraoral lesion with only a small intraoral component situated anterior to the tongue. There was no significant narrowing within the oral cavity or extension toward the pharynx, and the pharynx, larynx, trachea, and esophagus appeared intact. The tongue was normal, and cine MRI demonstrated preserved fetal swallowing. Taken together, these findings were consistent with a low anticipated risk of critical airway obstruction at delivery and suggested that postnatal airway establishment would likely be feasible without continued placental support. Delivery was therefore scheduled by elective cesarean section at 38 weeks in a tertiary setting, with obstetric, neonatology, pediatric anesthesia, and maxillofacial surgery teams present to permit immediate airway assessment and rescue intervention if required. These imaging findings are in line with previous reports on Neumann tumors and highlight several clinically relevant aspects, particularly regarding airway management and the timing of surgery. NT lesions may extend extra- or intraorally and may cause clinically significant airway compromise [7,8]. This risk is potentially life-threatening and should always be taken into consideration, especially when a life-saving intubation or even tracheotomy of the newborn may be required after delivery. The lesion itself can be surgically removed immediately after birth or within 24–72 h; however, timing largely depends on several factors, including the absence of worrisome bleeding, lack of airway compromise, stable general health of the mother, no restriction in breathing or feeding of the newborn, and the absence of pathological vestibular adhesions of the oral mucosa. Another important aspect when examining the newborn is to assess the extent and diameter of the epulis’ attachment to the oral cavity, as this greatly influences the scope of surgery. Butt et al., along with others [9,10], indicated that the shape and size of these lesions may vary, and some of them may cause airway compromise; rarely, fusion and atresia of nostrils and gums may also be present, which requires immediate surgical intervention. In most cases, a single epulis with well-organized and well-defined borders is found. Before any surgery is scheduled, serious questions should include the following: Is the lesion located to one anatomical area (lips or gingivae)? Does it spread in two or more anatomical areas? Is the bone, skin or muscles involved? Is it placed on vital structures such as the tongue or floor of the mouth? What nerve and vascular proximity is adjacent to the area? And is the scope of any possible vascular components and lesion vascularity known? As shown above, the condition of the lesion base affects decision-making ahead of surgery.

**Figure 4:**
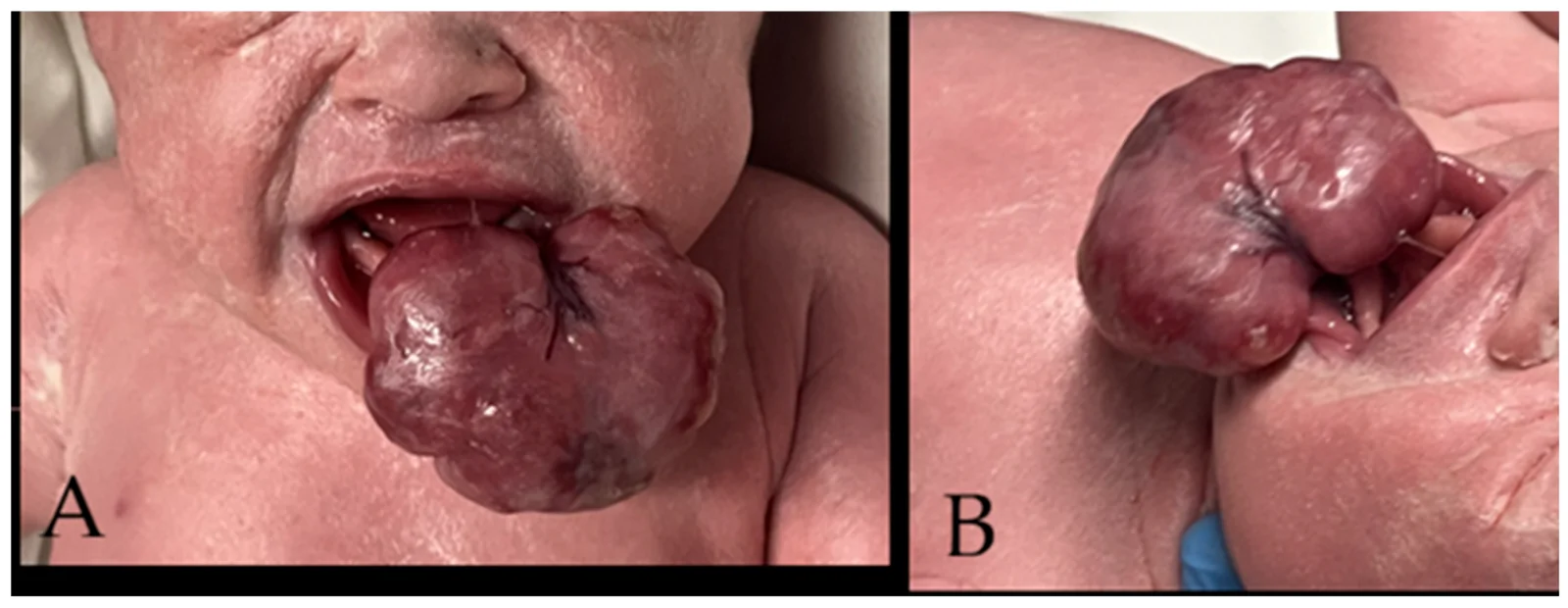
The newborn was delivered at 38 weeks of gestation. Delivery was scheduled as an elective cesarean section at 38 weeks of gestation with a multidisciplinary team present. The team included an obstetrician–gynecologist, an anesthesiologist, a neonatologist, a pediatric anesthesiologist, and maxillofacial surgeons. The female patient was born with a birth weight of 2900 g and an Apgar score of 9/10. Her cardiorespiratory adaptation was normal. No immediate airway intervention was required. After birth, the clinically visible exophytic component measured approximately 16 × 13 × 12 mm within the oral opening. No clinically evident pulsatility or active bleeding was observed. The upper lip shows no deviation. The lesion cannot be clearly separated from the lower lip and does not appear to be connected to the tongue. Postnatal examination and subsequent surgery confirmed that the lesion was attached to the mandibular alveolar ridge, with predominant extraoral protrusion between the lips. Prenatal USG performed at 32 weeks of gestation demonstrated a 22 × 19 × 17 mm homogeneously hypoechoic solid mass with moderate vascularity between the upper and lower lips. Because prenatal USG/MRI and postnatal clinical measurements were obtained using different methods and imaging planes, these dimensions should not be interpreted as directly comparable. In the newborn, the lesion appears independent of the upper lip; however, it cannot be reliably distinguished from the lower lip. Its possible extension toward the pharynx cannot be determined. Improved outcomes were achieved because of a combination of USG and MRI diagnostic approaches for further assessment and surgical planning. The extent of each exophytic soft-tissue gingival lesion should always be carefully assessed after birth to evaluate its vascular supply and borders. If the lesion extends toward the floor of the mouth or the tongue, resection with wider surgical margins may be required. Care should be taken to spare the floor of the mouth and salivary puncta. During initial evaluation of the newborn, upper airway patency and the lack of any anatomical obstacles in the nasal cavity, as well as the oropharynx, should be carefully evaluated. The lesion itself can appear as a round, exophytic tissue mass or have a granular, lobular consistency with irregularities or even bleeding areas. The presented NT appeared as a dark-reddish, smooth, soft, and slightly warm mass on clinical examination. The initial suspicion of NT is based on clinical evaluation and its typical characteristic features. Most commonly, the mass is well organized and soft on examination and exhibits clear borders, where the lips and oral commissure are not fused with the NT (**A**). Intraorally, the alveolar bone consistency and shape have to be visualized and palpated if no neonatal teeth or bone irregularities are present, as well as the base of the gingival epulis fusion with the mucosa of the alveolar body, with or without its spread towards the oral vestibule or oral cavity floor (**B**). Presented NT should be differentiated from other lesions, such as congenital soft tissue malformations, dermoid cysts, hemangioma, lymphatic malformations, melanotic or pigmented neuroectodermal tumors, or even rarely some odontogenic tumors or RMS (rhabdomyosarcoma) [5,6,7,8,9,10]. Initial extraoral evaluation excluded any significant pulsatile lesion or worrisome bleeding, while the rest of the oral cavity structures were not fused with the NT. Some studies and authors hypothesize [4,5,6,7,8,9,10,11,12] that the following lesions might be associated with tooth buds and other teeth-related tissues: remnants of dental papilla; possible extra-bone-related position of a dental follicle; and their possible relation with rare and atypical granular lesions, like granular cell ameloblastoma (GCA) or peripheral granulation lesion similar to peripheral ameloblastoma-like lesion or even other granular-cell lesions [13,14,15,16]. A detailed microscopic evaluation of the excised specimen is always mandatory in order to exclude the occurrence of any other worrisome tumors. Other differential diagnoses should include granular lesions of the lips, buccal mucosa, palate, or tongue, which may also be encountered; however, some smaller lesions may resolve spontaneously over time [17,18,19,20,21,22,23].

**Figure 5:**
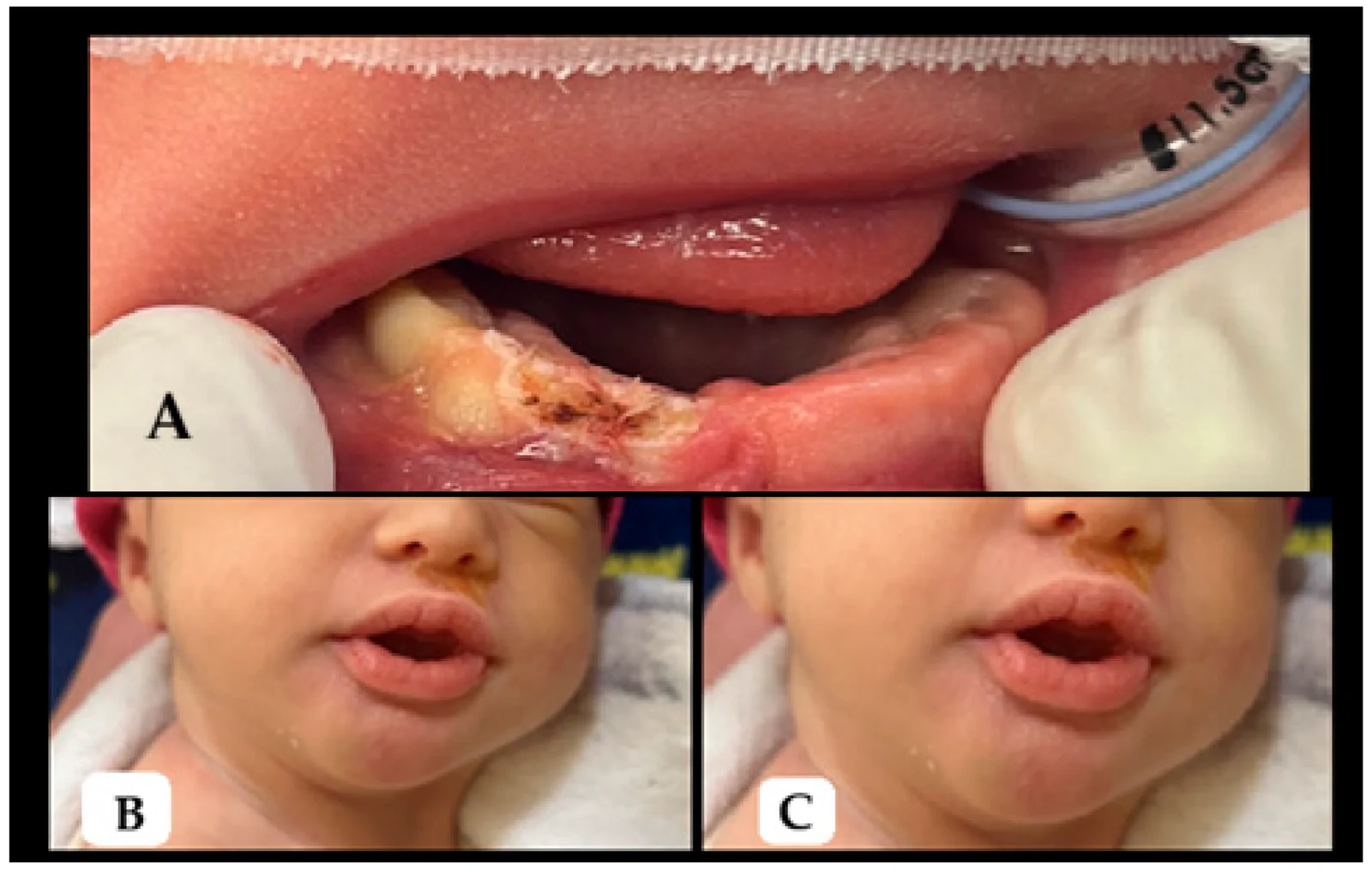
Within 24 h after birth, the baby was operated on under oro-tracheal general anesthesia. A step-by-step dissection with electrocautery was done. The lesion itself was excised without damaging any adjacent gingival or intraoral sutures. Under clear vision after retracting the lips, the pedicle of the lesion was ligated and cut by electrocautery. After meticulous hemostasis, the gingival structures were sutured with resorbable materials (Vicryl 5/0). Placed sutures should be resorbable and firmly attached to the gingival area to cover the bone deficit, if present. The specimen was submitted for histopathological examination. Final inspection of the gingival mucosa and mandibular vestibule demonstrated a satisfactory postoperative surgical result (**A**). The 7-day postoperative photograph demonstrates an excellent surgical outcome, with restored oral cavity patency, no visible swelling, adequate lip closure, and harmonious facial proportions (**B**,**C**). Additional surgical assessment should include the floor of the mouth, tongue, soft palate, and upper airway. Complete surgical excision remains the treatment of choice [10,11,12,13,14,15,16]. It is worth pointing out any other possible oral cavity anomalies that should be considered for differential diagnoses [20,21,22,23,24,25]. According to Zemet et al., magnetic resonance plays a pivotal role in differential diagnostics during the prenatal period, especially because of high-resolution detail evaluation, and is complementary in oral cavity, head, and neck evaluation [26]. Because of the combined effort of USG and MRI, many lesions can be investigated, and they should include the following differential diagnoses from selected oral, face, and head lesions: (1) cysts of the major and minor salivary glands; (2) thyroglossal duct cysts; (3) focal macroglossia; (4) teratomas (epignathus); (5) benign lesions such as lymphangioma, hemangioma, melanotic neuroectodermal tumor of infancy; (6) choristomas (lingual thyroid, glial, lingual sebaceous lesions, and more); or even (7) malignant lesions such as malignant melanoma, rhabdomyosarcoma, or spindle cell carcinoma [15,16,17,18,19,20,21,22,23,24]. In neonates with a prenatally identified oral mass and a potential risk of upper airway obstruction, delivery should be preceded by a predefined multidisciplinary difficult-airway management plan. Neonatology, pediatric anesthesia, and an experienced ENT/airway surgical team should be immediately available, together with appropriate equipment for face-mask ventilation, videolaryngoscopy, endotracheal intubation, flexible or rigid endoscopy, supraglottic airway placement, and emergency surgical airway access. Immediately after birth, airway patency, spontaneous breathing, and heart rate should be assessed, and effective ventilation established according to neonatal resuscitation principles. If face-mask ventilation is ineffective, airway position and mask seal should be optimized, and suction should be performed if mechanical obstruction by secretions is suspected. Repeated unsuccessful intubation attempts should be avoided, with early escalation to videolaryngoscopy or endoscopic airway management. If tracheal intubation cannot be achieved, a supraglottic airway may be considered when anatomically feasible, while flexible or rigid endoscopic intubation and emergency tracheostomy should be available as rescue options. In cases in which secure postnatal airway establishment is considered unlikely or potentially unsafe because of the size or anatomical relationship of the mass, delivery on placental support using an EXIT strategy should be considered.

**Figure 6:**
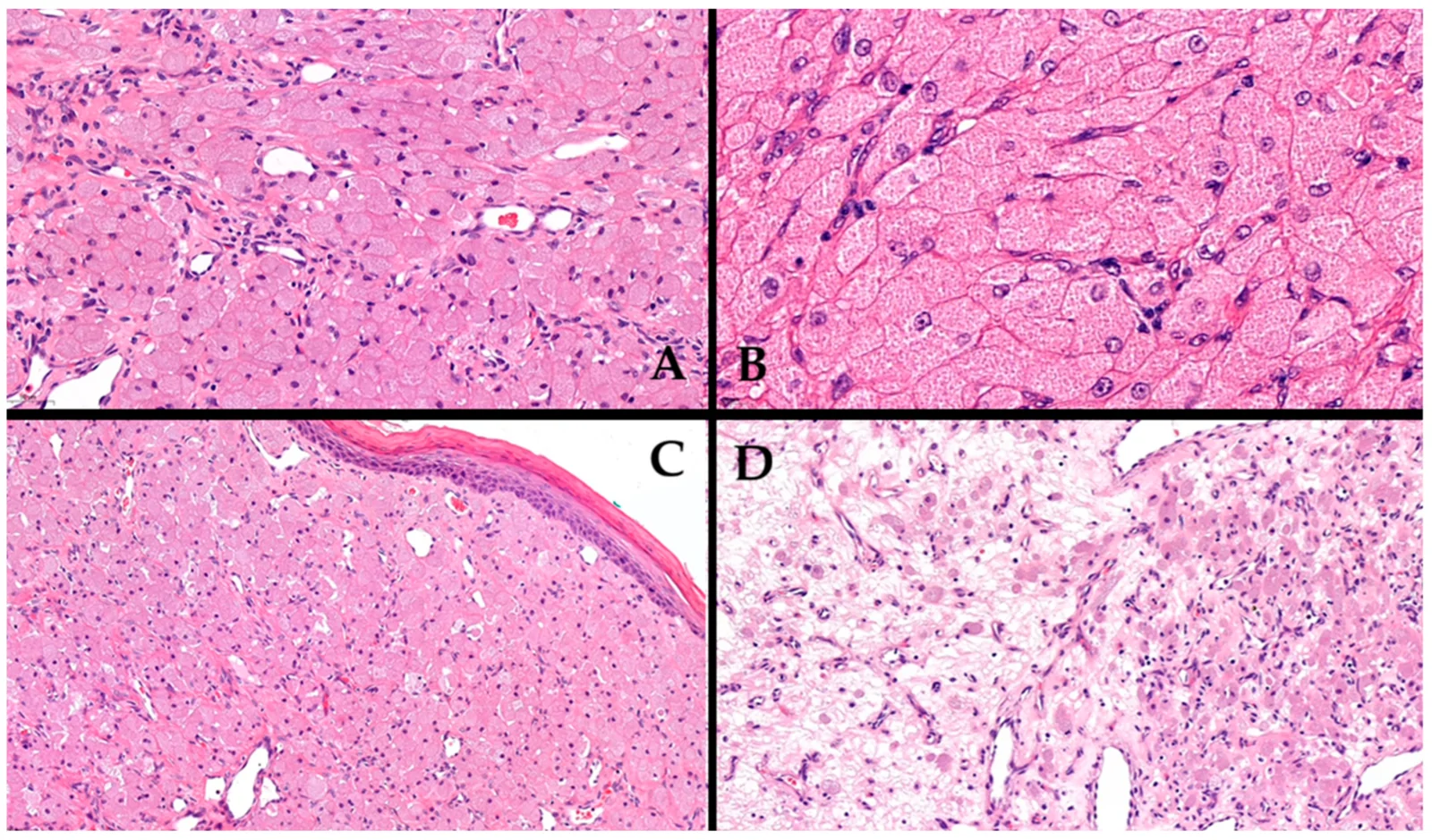
A well-circumscribed soft tissue nodule excised from the alveolar ridge of the mandible of a 1-day-old child was submitted for histopathological examination. The specimen was fixed in 10% neutral buffered formalin, routinely processed, embedded in paraffin, and sectioned at 3–4 µm. Slides were stained with hematoxylin and eosin (H&E). Due to the characteristic morphological appearance, no additional special stains or immunohistochemical studies were performed. The histopathological description notes: The sections reveal a well-demarcated, non-encapsulated, polypoid lesion located within the submucosal stroma. Image showing the cells present in the lesion along with the accompanying capillaries (**A**). Visible polymorphic cells containing granular, strongly eosinophilic cytoplasm (**B**). The nuclei are small, round, and uniform, with inconspicuous nucleoli. Visible border of the tumor covered with keratinizing stratified squamous epithelium. The adjacent mucosa shows no evidence of hyperplasia, ulceration, or dysplasia (**C**). Visible edematous, richly vascularized stroma of a repair nature (**D**). No necrosis, hemorrhage, or infiltrative growth pattern is observed. No features of malignancy are present. Clinically, NT appears as a smooth, sessile, or pedunculated mass that may interfere with feeding or respiration in neonates, prompting surgical removal. Histologically, NT is characterized by large granular cells with abundant eosinophilic cytoplasm and uniform nuclei arranged in sheets or nests. In the literature, NT is generally reported to be negative for S100 protein, which may help distinguish it from other granular cell lesions [12,13,14,15,16]. No immunohistochemical staining was performed in the present case because the morphology was considered characteristic. In some cases, improved diagnostics with endoscopic evaluation of the nose, throat, pharynx, and adjacent areas should be performed simultaneously when the newborn is under general anesthesia to support decision-making [17,18]. Each excised specimen should always be assessed histopathologically to exclude other worrisome conditions [19,20]. The presented NT case suggests that a characteristic histopathological profile can confirm the initial diagnosis; therefore, a good histopathological examination is the key to avoiding any misdiagnosis [24,25,26]. In summary, both USG and MRI improve the diagnostics of any tumor within the oral cavity and facial skeleton and should be used in a combined effort, especially in addressing each lesion’s size, boundaries, adjacent tissues, and its possible vascular components or pathological spread within its borders. Furthermore, the location of the lesion solely in soft tissues, or its spread to the bone, should be noted. The structure of soft, solid, or mixed mass appearances can also improve the final prenatal diagnostics and prepare each clinician for the next step after birth.

## Data Availability

The datasets used and/or analyzed during the current study are available from the corresponding author upon reasonable request.
